# BAY 81-8973 Efficacy and Safety in Previously Untreated and Minimally Treated Children with Severe Hemophilia A: The LEOPOLD Kids Trial

**DOI:** 10.1055/s-0042-1757876

**Published:** 2023-01-10

**Authors:** Rolf Ljung, Anthony K. C. Chan, Heidi Glosli, Olubunmi Afonja, Bastian Becker, Despina Tseneklidou-Stoeter, Maria Elisa Mancuso, Sonata Saulyte-Trakymiene, Gili Kenet

**Affiliations:** 1 Department of Clinical Sciences Lund-Pediatrics, Lund University, Lund, Sweden; 2 McMaster Children's Hospital, McMaster University, Hamilton, Ontario, Canada; 3 Centre for Rare Disorders and Department of Pediatric Research, Oslo University Hospital, Oslo, Norway; 4 Bayer, Whippany, New Jersey, United States; 5 Bayer, Wuppertal, Germany; 6 Bayer, Berlin, Germany; 7 Center for Thrombosis and Hemorrhagic Diseases, IRCCS Humanitas Research Hospital, Rozzano, Milan, Italy; 8 Clinic of Children's Diseases, Faculty of Medicine, Vilnius University Hospital Santaros Klinikos, Vilnius University, Vilnius, Lithuania; 9 Sheba Medical Center, The Israeli National Hemophilia Center, The Amalia Biron Thrombosis Research Institute and The Sackler Medical School, Tel Aviv University, Tel-Hashomer, Israel

**Keywords:** immune tolerance induction, clinical trial, factor VIII, hemophilia A, inhibitors, prophylaxis

## Abstract

**Introduction** BAY 81–8973, a full-length recombinant factor VIII for hemophilia A treatment, has been extensively evaluated in previously treated patients in the LEOPOLD (Long-Term Efficacy Open-Label Program in Severe Hemophilia A Disease) clinical trials.

**Aim** To assess BAY 81–8973 efficacy and safety when used for bleed prophylaxis and treatment in previously untreated/minimally treated patients (PUPs/MTPs).

**Methods** In this phase III, multicenter, open-label, uncontrolled study, PUPs/MTPs (<6 years old) with severe hemophilia A received BAY 81–8973 (15–50 IU/kg) at least once weekly as prophylaxis. Primary efficacy endpoint was the annualized bleeding rate (ABR) within 48 hours after prophylaxis infusion. Adverse events and immunogenicity were assessed. Patients who developed inhibitors were offered immune tolerance induction (ITI) treatment in an optional extension phase.

**Results** Fifty-two patients were enrolled, with 43 patients (mean age: 13.6 months) treated. Median (interquartile range) ABR for all bleeds within 48 hours of prophylaxis infusion was 0.0 (0.0–1.8) among patients without inhibitors ( *n* = 20) and 0.0 (0.0–2.2) among all patients. As expected, inhibitors were the most frequent treatment-related adverse event (high titer: 17 [39.5%] patients; low titer: 6 [13.9%] patients). Six of 12 patients who underwent ITI treatment in the extension phase (high titer [ *n* = 5], low titer [ *n* = 1]) achieved a negative inhibitor titer.

**Conclusion** BAY 81–8973 was effective for bleed prevention and treatment in PUPs/MTPs. The observed inhibitor rate was strongly influenced by a cluster of inhibitor cases, and consequently, slightly higher than in other PUP/MTP studies. Overall, the BAY 81–8973 benefit–risk profile remains unchanged and supported by ongoing safety surveillance. Immune tolerance can be achieved with BAY 81–8973.

## Graphical abstract

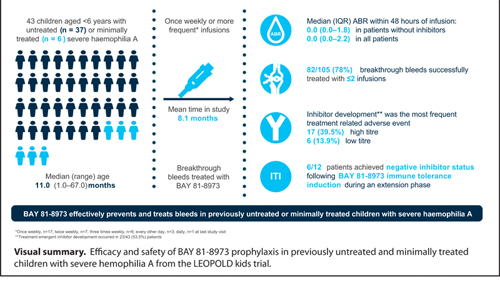

## Introduction

Patients with severe hemophilia A (factor VIII activity [FVIII:C] <1 IU/dL) typically experience spontaneous bleeds, primarily into joints. 1 Over time, repeated bleeds into the same joint trigger progressive damage that can develop into hemophilic arthropathy, resulting in pain, deformity, and disability. 2 The clinical management of patients with severe hemophilia A is therefore based upon prevention of bleeding and prompt treatment of bleeds to prevent joint damage. 1

Currently, the main treatment and standard of care for preventing bleeds in hemophilia is regular prophylaxis with FVIII replacement therapy. 1 The benefits of prophylaxis in this setting have been recognized for over half a century, 3 and existing evidence shows that prophylaxis regimens can slow the progression of established joint disease 4 5 6 7 8 and even prevent joint damage from developing if initiated at an early age. 3 8 9 10 Indeed, the timing of prophylaxis initiation is a strong predictor of joint outcomes, 1 with early initiation (before the age of 2.5 years) providing better protection against joint damage throughout childhood and adolescence than delayed initiation (after the age of 6 years). 7 11 Accordingly, current guidelines recommend starting prophylaxis prior to the onset of joint disease and before the age of 3 years. 1

The most serious and clinically significant complication associated with all FVIII concentrates is the development of anti-FVIII antibodies (inhibitors) that neutralize the function of infused FVIII concentrates, 1 12 rendering FVIII replacement therapy ineffective and causing substantial morbidity, mortality, and reduced quality of life. 1 13 14 15 As inhibitors usually develop within the first 50 exposure days (EDs) after beginning FVIII replacement therapy, 16 previously untreated patients (PUPs) are particularly susceptible to inhibitor development.

BAY 81–8973 (Kovaltry ^®^ , octocog alfa; Bayer, Berkeley, California, United States) is a full-length, unmodified, recombinant, human FVIII indicated for the treatment and prophylaxis of bleeding in patients of all ages with hemophilia A. With the same primary amino acid sequence as sucrose-formulated recombinant FVIII (rFVIII-FS; Kogenate ^®^ FS, Bayer), BAY 81–8973 is produced using an enhanced manufacturing process that eliminates human- and animal-derived raw materials, increases pathogen safety, and ensures a consistently high degree of sialylation of N-linked glycans on the molecular surface—a posttranslational modification important for the half-life of some mammalian proteins, including glycoproteins. 17 18

The current study is part of the Long-Term Efficacy Open-Label Program in Severe Hemophilia A Disease (LEOPOLD) clinical trial program, which investigated the pharmacokinetics, efficacy, and safety of BAY 81–8973 in patients with severe hemophilia A. The LEOPOLD I and LEOPOLD II trials showed that prophylaxis with BAY 81–8973 two or three times per week was effective for the treatment and prevention of bleeds in previously treated adult and adolescent patients (≥12 years old). 19 20 Previously treated children aged ≤12 years with severe hemophilia A received BAY 81–8973 prophylaxis (≥2 times per week) in the LEOPOLD Kids Part A clinical study; overall, the median annualized bleeding rate (ABR) was 1.9 for total bleeds and 0.0 for joint bleeds, and 90% of bleeding episodes required ≤2 infusions. 21 In all of these studies, conducted in previously treated patients (PTPs) with no current or prior history of inhibitors, BAY 81–8973 demonstrated good tolerability, and no patients developed inhibitors.

Here, we present the efficacy and safety results of the LEOPOLD Kids Part B clinical study, conducted to assess the pharmacokinetics, efficacy, and safety of BAY 81–8973 for prophylaxis and treatment of bleeds in previously untreated and minimally treated children with severe hemophilia A; immune tolerance induction (ITI) results from the optional extension phase are also included for those patients who entered it to start ITI treatment.

## Methods

### Study Design

**Fig. 1:**
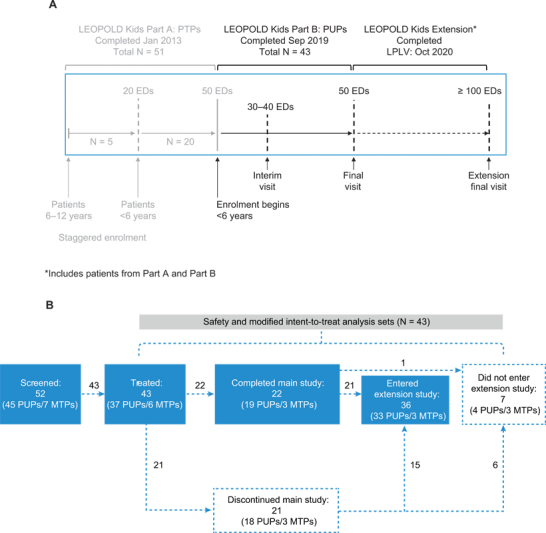
**(A)** The LEOPOLD Kids study: enrolment timeline. Note: LEOPOLD Kids Part A and Part B ran in parallel, with enrolment for Part B beginning when 20 PTPs from Part A had reached 50 EDs. Only Part B of the main study is reported in this article. Part A has been previously reported, 21 and the extension phase will be reported separately. **(B)** LEOPOLD Kids Part B: patient disposition. Note: The extension phase will be reported separately. EDs, exposure days; LPLV, last patient last visit; MTPs, minimally treated patients; *N* , number of patients; PTPs, previously treated patients; PUPs, previously untreated patients.

LEOPOLD Kids comprised two parts: Part A was conducted in PTPs aged ≤12 years, 21 while Part B was conducted in PUPs and minimally treated patients (MTPs) aged <6 years. The demonstration of safety in at least 20 PTPs who received a minimum of 50 EDs in Part A was a necessary requirement to start enrolment in Part B, and patients enrolled into Part B were to continue in the study until they reached 50 EDs ( Fig. 1A ). Patients from both Part A and Part B were offered participation in an optional open-label extension phase, allowing observation for a total of ≥100 EDs. Patients who developed inhibitors during the main study of Part B also had the option to roll over into the optional extension phase to start ITI treatment before completing the main study. Parts A and B of the study have been completed, and Part A of the main study has been previously reported 21 ; the current article reports results from Part B of the main study, and also includes the results from the optional extension phase for those patients who entered it to start ITI.

LEOPOLD Kids Part B was a multicenter, open-label, uncontrolled, phase III study (ClinicalTrials.gov identifier: NCT01311648) conducted at 30 centers in 15 countries between September 2012 and September 2019. All patients received BAY 81–8973 (15–50 IU/kg; minimum dose: 250 IU) for prophylaxis at least once weekly; however, treatment could also be initiated with an on-demand regimen, as this provided guidance on when to initiate prophylaxis. Breakthrough bleeds were also treated with BAY 81–8973. Surgery (major or minor) could be performed under BAY 81–8973 coverage, with dosing following the standard practice used for Kogenate FS ^®^ /Kogenate ^®^ .

Patients who developed inhibitors and rolled over into the extension phase to begin ITI treatment received BAY 81–8973 for approximately 18 months at a dose of up to 200 IU/kg per day (or 100 IU/kg twice daily at the investigator's discretion) until successful inhibitor eradication or ITI failure. Treatment with ITI beyond 18 months required approval from the sponsor and the coordinating investigator.

The study protocol and amendments were reviewed and approved by each study site's independent ethics committee or institutional review board. The study was conducted in accordance with the Declaration of Helsinki and the International Council for Harmonisation Good Clinical Practice guidelines. Written informed consent was provided by parents or legal representatives.

### Patients

The study included children aged <6 years with severe hemophilia A (FVIII:C <1 IU/dL), no previous exposure to any FVIII product (PUPs), or ≤3 EDs to any FVIII product and no history or current evidence of FVIII inhibitors (MTPs).

Exclusion criteria were bleeding disorders other than hemophilia A, thrombocytopenia (platelet count <100,000/mm ^3^ ), creatinine level greater than twice the upper limit of normal or aspartate/alanine aminotransferase levels greater than five times the upper limit of normal, use of chemotherapy or immunomodulatory agents, use of another investigational FVIII product within the last month, use of another experimental drug within the last 3 months, requirement for premedication to tolerate FVIII treatment, and known hypersensitivity to active substance or mouse or hamster protein.

### Efficacy

The primary efficacy variable was the annualized number of total bleeds occurring within 48 hours after a prophylaxis infusion (sum of spontaneous, trauma-related, untreated, and other bleeds). This parameter was chosen due to the variable treatment intervals required in children, for whom a lower injection frequency may be needed due to venous access problems. 21 Secondary efficacy variables were the annualized number of total bleeds during prophylaxis treatment (sum of spontaneous, trauma-related, untreated, and other bleeds), hemostatic outcome of surgeries (blood loss, transfusion, and/or hemostasis-related complications), and FVIII recovery. Additional efficacy variables comprised the following: annualized number of total bleeds, joint bleeds (assessed by clinical judgment), spontaneous bleeds, and trauma-related bleeds overall; number of treatments required to control bleeds; patient/caregiver assessment of treatment response, categorized as excellent (abrupt pain relief and/or improvement in signs of bleeding, with no additional infusions administered), good (definite pain relief and/or improvement in signs of bleeding, but possibly requiring more than one infusion for complete resolution), moderate (probable or slight improvement in signs of bleeding, with at least one additional infusion for complete resolution), or poor (no improvement at all between infusions, or condition worsens); and FVIII utilization. When patients were identified as having a high-titer inhibitor, they withdrew from the main study and had the option to receive ITI in the extension phase at the investigator's discretion and caregiver's decision; therefore, efficacy data were collected up to the time when the inhibitor was confirmed.

Participants had site visits at screening, baseline (or combined screening and baseline for PUPs), and after they reached approximately 5 EDs (visit 3), 10 EDs (visit 4), 15 EDs (visit 5), 20 EDs (visit 6), and 50 EDs (final visit). Patients with <40 EDs by 6 months post-baseline also had an interim visit at 30 to 40 EDs. Bleeding and treatment information was recorded by parents/caregivers in electronic diaries.

### FVIII Incremental Recovery

Blood samples for assessment of recovery were collected preinfusion and 20 to 30 minutes postinfusion at baseline, visit 6 (20 EDs), and the final visit (50 EDs) in patients who were not actively bleeding. Preinfusion blood collection was to be performed ≥48 hours after the previous infusion of BAY 81–8973. Plasma FVIII:C was measured in the central laboratory using the chromogenic assay.

### Biomarker Evaluation

An exploratory biomarker analysis using the Sanger gene sequencing method 22 23 was conducted to identify markers for risk of inhibitor development. Participation was recommended but not mandatory. The markers analyzed included the type of *F8* gene pathogenic variants and *F8* gene benign variants. For *F8* gene mutations, those considered to be high risk for inhibitor development included large deletions, nonsense mutations, and intron-22/intron-1 inversions, while small deletions and insertions, missense mutations, and splice-site mutations are considered lower risk. 24 25 Inhibitor risk types were also associated with mutation type subgroups and location. 26

### Safety

Adverse events (AEs) and serious AEs (SAEs) were monitored at each study visit and assessed in terms of seriousness, severity, and relationship to study drug. Other safety evaluations included vital signs, which were assessed at every visit, and standard safety laboratory variables (complete blood count with differential and serum chemistry), assessed at screening/baseline and the final visit (50 EDs). The development of inhibitors (defined as ≥0.6 Bethesda units/milliliter [BU/mL]), as measured according to the Nijmegen-modified Bethesda assay at a central laboratory, was considered a SAE. Inhibitor assessment was conducted at screening and baseline for MTPs and then at every study visit thereafter for all patients.

### Statistical Analysis

The safety population included all patients who received at least one dose of BAY 81–8973. Efficacy data were analyzed in the modified intent-to-treat (mITT) and per protocol (PP) populations. The mITT population included all patients in the safety population who had infusion/bleeding data available. The PP population was defined as all ITT subjects who completed the study with no major protocol deviations. All efficacy and safety data were summarized using descriptive statistics and conducted for all treated patients, PUPs, and MTPs.

Efficacy data were also analyzed according to inhibitor status (without inhibitor; low-titer inhibitor; high-titer inhibitor) for the mITT and PP populations. Patients who developed a high-titer inhibitor (>5 BU) remained in the main study until the presence of inhibitor was confirmed by analysis of a second sample (performed in the central laboratory) within 2 weeks of the investigator's notification of initial high-titer inhibitor detection, at which point they withdrew from the main study to begin ITI treatment (with the option of entering the extension phase). Therefore, for high-titer inhibitor patients, data on bleeds and treatment efficacy were only collected and analyzed up to the time when inhibitors were confirmed. Patients who developed a confirmed low-titer inhibitor could be withdrawn from the main study to initiate ITI at the investigator's discretion, but otherwise they remained in the main study. Efficacy data for these patients were therefore collected for the entire time they remained in the main study and received prophylaxis (both before and after confirmation of low-titer inhibitor).

Statistical analyses were conducted using SAS version 9.2 (SAS Institute Inc., Cary, North Carolina, United States).

## Results

### Patients

Out of 52 patients enrolled (45 PUPs; 7 MTPs), 43 received at least one dose of the study drug and were included in the safety and mITT populations (37 PUPs; 6 MTPs) ( Fig. 1B ). Baseline demographics and clinical characteristics are shown in Table 1 . Major protocol deviations were identified in three patients (2 PUPs and 1 MTP); all were related to infusion of study treatment (missing information on study drug administration [ *n* = 3], including one patient with an interval of >30 days between infusions). Of the 43 patients treated in the main study, 22 (19 PUPs/3 MTPs) completed it (i.e., reached 50 EDs) and 21 discontinued early ( Fig. 1B ), with 18 (16 PUPs; 2 MTPS) discontinuing because of inhibitor development (17 patients had high-titer inhibitor, 1 patient had a low-titer inhibitor). Three patients discontinued due to miscalculation of EDs ( *n* = 1), incorrect visit planning ( *n* = 1), and logistical reasons (a vacation that prevented completion of main study; *n* = 1). Five patients who developed low-titer inhibitor remained in, and completed, the main study.

**Table 1:** Baseline demographics and disease characteristics

	PUPs ( *N* = 37)	MTPs ( *N* = 6)	Total ( *N* = 43)
Age, mo			
Mean (SD)	12.0 (5.3)	23.2 (23.2)	13.6 (10.2)
Median (range)	10.0 (2.0–33.0)	18.0 (1.0–67.0)	11.0 (1.0–67.0)
Race, *n* (%)			
White	34 (91.9)	3 (50.0)	37 (86.0)
Black	0	1 (16.7)	1 (2.3)
American Indian or Alaska native	0	1 (16.7)	1 (2.3)
White, American Indian, or Alaska native	1 (2.7)	0	1 (2.3)
Not reported	2 (5.4)	1 (16.7)	3 (7.0)
Age at diagnosis, mo			
Number of evaluable patients a	34	3	37
Mean (SD)	6.6 (7.0)	8.0 (1.0)	6.7 (6.7)
Median (range)	6.0 (0.0–32)	8.0 (7.0–9.0)	7.0 (0.0–32.0)
Presence of target joints b , *n* (%)			
Yes	1 (2.7)	1 (16.7)	2 (4.7)
No	36 (97.3)	5 (83.3)	41 (95.3)
Number of target joints b , *n* (%)			
0	36 (97.3)	5 (83.3)	41 (95.3)
1	0	1 (16.7)	1 (2.3)
2	1 (2.7)	0	1 (2.3)
Number of bleeds in the period up to 12 months prior to enrolment c			
Number of evaluable patients a	36	6	42
Mean (SD)	0.9 (1.5)	2.2 (1.6)	1.1 (1.6)
Median (range)	0.0 (0.0–7.0)	1.5 (1.0–5.0)	1.0 (0.0–7.0)
Number of joint bleeds in the 12 months prior to enrolment c			
Number of evaluable patients a	36	6	42
Mean (SD)	0.0 (0.2)	0.2 (0.4)	0.1 (0.2)
Median (range)	0.0 (0.0–1.0)	0.0 (0.0–1.0)	0.0 (0.0–1.0)

Abbreviations: MTPs, minimally treated patients; *N* , total number of patients; *n* , number of patients; PUPs, previously untreated patients; SD, standard deviation.

a Data were not available for some patients.

b Presence of target joints was reported by the investigator at baseline.

c The observation period for number of bleeds and joint bleeds occurring within 12 months prior to enrolment is based on patient age, ranging from 1 month (in the youngest subject, who was 1 month old at study entry) to 12 months (for all subjects who were >12 months old at study entry).

A total of 36/43 patients from the main study (20 with inhibitors [14 with high-titer inhibitor, 6 with low-titer inhibitor], and 16 patients without inhibitor) entered the extension phase. Out of the 20 patients with inhibitors who entered the extension phase, 12 received ITI treatment (11 patients with high-titer inhibitor and 1 with low-titer inhibitor), the results of which will be reported here; results for the other patients who entered the extension phase will be reported separately.

### Extent of Exposure

The mean (±standard deviation [SD]) time in the main study was approximately 8.1 (5.0) months for the whole population, 8.5 (5.1) months for PUPs, and 5.3 (3.4) months for MTPs. The median (range) number of EDs among patients in the entire mITT population (i.e., all patients, including those who developed inhibitor and those who did not) was 46 (1–55; 11 patients had accumulated >50 EDs at the time of end of study visit for the main study); among PUPs and MTPs, median (range) EDs were 46 (6–55) and 36.5 (1–55), respectively. The sums of all EDs were 1,488, 1,303, and 185 for the total population, PUPs, and MTPs, respectively.

Of the 20 patients who did not have a documented positive inhibitor test ( *n* = 20) during the main study, 17 completed the study, with 8 having accumulated >50 EDs at the time of end of main study visit for the main study; three patients had <10 EDs with BAY 81–8973 prior to leaving the main study.

In total, 1,586 infusions of BAY 81–8973 were administered during the main study, 1,243 of which were prophylactic doses (303 doses were given on-demand and 40 were administered for other reasons, e.g., surgery). At the last study visit, 17 patients were prescribed a dosing frequency of once weekly and 17 patients were prescribed a dosing frequency of at least twice weekly (twice weekly, *n* = 7; three times weekly, *n* = 6; every other day, *n* = 3; daily, *n* = 1) for prophylaxis. The median (range) number of prophylaxis doses in all patients was 34.5 (1–55), while the mean (SD) number of prophylaxis doses was 29.6 (18.8). The mean (SD) nominal dose per prophylaxis infusion was 31.1 (9.3) IU/kg (median: 29.1 [range: 9–50] IU/kg).

### Efficacy

#### Annualized Number of Total Bleeds within 48 Hours after a Prophylaxis Infusion

The annualized number of total bleeds occurring within 48 hours after a prophylaxis infusion is summarized in Table 2 . When considering only those patients who did not develop an inhibitor at any time during the study ( *N* = 20), 12 (60%) did not experience a bleed and 8 (40.0%) experienced 10 bleeds within 48 hours of a prophylaxis infusion. Accordingly, the median (interquartile range [IQR]) ABR for bleeds within 48 hours after prophylaxis infusion in patients who did not develop inhibitors was 0.0 (0.0–1.8), and the respective mean (SD) ABR was 0.9 (1.4) ( Table 2 ).

**Table 2:** Total bleeds within 48 hours after prophylaxis infusion by inhibitor status

	Inhibitor status		
	No inhibitors ( *N* = 20)	Low titer a ( *N* = 6)	High titer b ( *N* = 17)
Patients with at least one bleed within 48 hours after prophylaxis infusion, *n* (%)			
No	12 (60.0)	4 (66.7)	9 (52.9)
Yes	8 (40.0)	2 (33.3)	8 (47.1)
ABR for total bleeds occurring within 48 hours after prophylaxis infusion			
Median (IQR)	0.0 (0.0–1.8)	0.0 (0.0–5.0)	0.0 (0.0–4.9)
Mean (SD)	0.9 (1.4)	3.1 (5.4)	2.8 (3.7)

Abbreviations: ABR, annualized bleeding rate; IQR, interquartile range; *N* , total number of patients; *n* , number of patients; SD, standard deviation.

a Patients who developed low-titer inhibitors remained in the study, apart from one patient who was removed at the investigator's discretion to initiate ITI in the extension phase. Therefore, the presented bleed data represent the entire time on prophylaxis (before and after confirmation of low-titer inhibitors).

b All patients who developed a high-titer inhibitor were removed from the study to initiate ITI (with the option of entering the extension phase). Therefore, bleed data for patients who developed high-titer inhibitors represent *only* the period before inhibitors were confirmed.

In the entire mITT ( *N* = 43) and PP ( *N* = 40) populations, the median (IQR) (mean [SD]) ABRs for total bleeds occurring within 48 hours after a prophylaxis infusion were 0.0 (0.0–2.2) (1.9 [3.3]) and 0.0 (0.0–3.0) (2.1 [3.3]), respectively. Among patients with no inhibitor or low-titer inhibitor ( *N* = 26), the corresponding median (IQR) (mean [SD]) ABR was 0.0 (0.0–1.9) (1.4 [2.9]).

In the overall population, the median (IQR) ABR for all bleed types (spontaneous, trauma and joint bleeds) occurring within 48 hours after a prophylaxis infusion was 0.0 (0.0–0.0). A summary of bleeds occurring within 48 hours after a prophylaxis infusion is presented according to bleed type and inhibitor status in Supplementary Table S1 (available in the online version). Similar data were observed for the PP population ( Supplementary Table S2 [available in the online version]).

As patients who developed low-titer inhibitors remained in the study (unless withdrawn at the investigator's discretion), ABRs for total bleeds occurring within 48 hours after a prophylaxis infusion were also evaluated during the period in which low-titer inhibitors were present in these patients. These data are summarized in Supplementary Table S3 (available in the online version).

#### Annualized Number of Bleeds

While the primary efficacy variable limited the interval of interest to the first 48 hours after a prophylaxis infusion, further efficacy variables were analyzed independent of time of prophylaxis infusion. Overall, 184 bleeds occurred during the study period, with 86.0% of patients experiencing ≥1 bleed. Trauma-related bleeds were the most frequent bleed type, followed by spontaneous and joint bleeds (62, 42, and 25 bleeds, respectively).

A summary of bleeds reported at any time during the study in the mITT population is presented according to inhibitor status in Table 3 , and bleeds reported during the total period of low-titer inhibitor in patients who developed low-titer inhibitors are summarized in Supplementary Table S3 (available in the online version). Bleeds were reported in 16/20 (80.0%) patients who did not develop an inhibitor, at a median (IQR) rate of 3.5 (1.5–7.4) total bleeds per year (mean [SD]: 5.3 [5.8]). Traumatic bleeds were more frequent in this patient group ( Table 3 ). One PUP, who did not develop an inhibitor, had 29 bleeds, approximately half of which occurred before the start of prophylaxis; all of these bleeds were mild or moderate in severity and 28 bleeds did not require treatment. There were no central nervous system or life-threatening bleeds. Bleeds were reported in 33/40 (82.5%) patients in the PP population, with a median (IQR) ABR for total bleeds of 4.7 (2.2–9.0); the respective mean (SD) ABR was 7.4 (8.9). A summary of bleeds by inhibitor status in the PP population is presented in Supplementary Table S2 (available in the online version).

**Table 3:** Annualized bleeding rate by bleed type and inhibitor status

	Inhibitor status		
	No inhibitors ( *N* = 20)	Low titer a ( *N* = 6)	High titer b ( *N* = 17)
Patients with ≥1 bleed, *n* (%)			
No	4 (20.0)	0.0	2 (11.8)
Yes	16 (80.0)	6 (100.0)	15 (88.2)
ABR for total bleeds			
Median (IQR)	3.3 (1.5–7.4)	5.9 (4.3–16.2)	7.4 (4.3–8.9)
Range	0–18	1–19	0–45
Mean (SD)	5.0 (4.9)	8.8 (7.1)	9.8 (11.2)
ABR for spontaneous bleeds			
Median (IQR)	0.0 (0.0–1.4)	2.5 (0.0–4.6)	0.0 (0.0–2.4)
Range	0–9	0–13	0–11
Mean (SD)	0.9 (2.1)	3.8 (5.0)	2.4 (3.9)
ABR for traumatic bleeds			
Median (IQR)	2.1 (0.0–3.2)	1.4 (0.0–2.2)	3.4 (0.0–5.7)
Range	0–9	0–4	0–45
Mean (SD)	2.4 (2.5)	1.5 (1.4)	5.6 (10.5)
ABR for joint bleeds			
Median (IQR)	0.0 (0.0–1.5)	0.7 (0.0–1.9)	0.0 (0.0–1.4)
Range	0–4	0–5	0–7
Mean (SD)	0.9 (1.2)	1.3 (1.8)	1.2 (2.0)

Abbreviations: ABR, annualized bleeding rate; IQR, interquartile range; *N* , total number of patients; *n* , number of patients; SD, standard deviation,

a Patients who developed low-titer inhibitors remained in the study, apart from one patient who was removed at the investigator's discretion to initiate ITI in the extension phase. Therefore, the presented bleed data represent the entire time on prophylaxis (before and after confirmation of low-titer inhibitors).

b All patients who developed a high-titer inhibitor were removed from the study to initiate ITI (with the option of entering the extension phase). Therefore, bleed data for patients who developed high-titer inhibitors represent *only* the period before inhibitors were confirmed.

#### Treatment of Bleeds

The majority of bleeds during the prophylaxis period (∼97%) were mild or moderate in severity, and most bleeds treated with BAY 81–8973 (82/105 [78%]) required ≤2 infusions; 23 (21.9%) bleeds required ≥3 infusions (12 of these bleeds occurred in patients who were eventually found to have a high-titer inhibitor, and 11 occurred in patients with no inhibitors or who were eventually confirmed to have low-titer inhibitors). Overall, the median (range) nominal dose administered to treat bleeds was 40.5 (21–112) IU/kg. Patient/caregiver response to treatment was reported as “good” or “excellent” in 79.0% of assessed bleeds in the mITT population, and in 93.5% of assessed bleeds in patients who did not develop an inhibitor during the study. An additional four bleeds occurred in patients who developed inhibitors and were treated with bypassing agents.

### Surgery

Five minor surgeries (one simple frenectomy and four port insertions, including one with external jugular vein cutdown) were performed. Hemostasis was rated “good” or “excellent” in all four minor surgeries for which assessment was available ( Supplementary Table S4 (available in the online version)). Data on the timing of BAY 81–8973 infusions were available for three minor surgeries: in one procedure (simple frenectomy), one dose of BAY 81–8973 was administered only on the day of surgery; for the remaining two procedures (each done in two patients with high-titer inhibitor), BAY 81–8973 was administered in 11 doses given over 8 days (port placement) and in eight doses over 4 days (external jugular vein cutdown and port placement), beginning on the day of surgery.

### FVIII Incremental Recovery

Measurement of FVIII recovery was performed in conjunction with planned prophylaxis infusions using the patient's usual prophylaxis dose (these analyses excluded invalid data). The mean (SD) incremental recovery for patients who did not develop inhibitors and who had recovery data available ( *N* = 17) was 1.76 (0.55) IU/dL per IU/kg.

### Safety

**Fig. 2:**
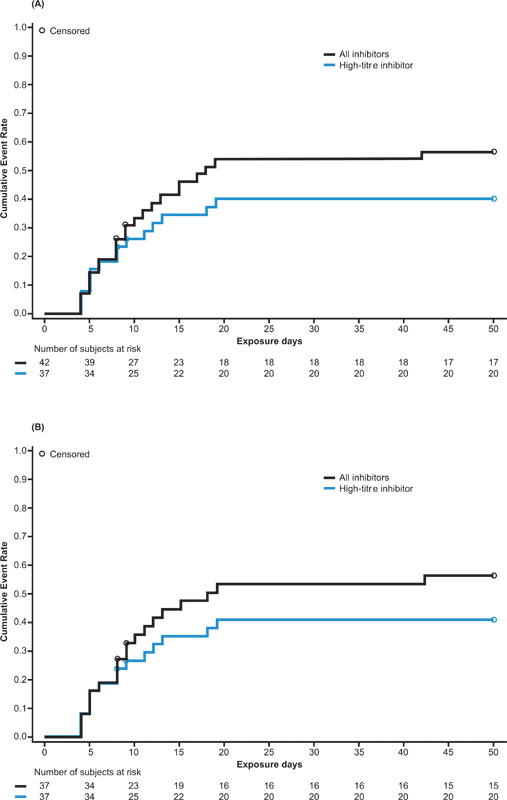
Kaplan–Meier survival curves showing development of inhibitors in **(A)** the overall study population ( *n* = 43 [37 PUPs, 6 MTPs]) and **(B)** PUPs only ( *n* = 37). MTPs, minimally treated patients; PUPs, previously untreated patients.

Treatment-emergent AEs (TEAEs; AEs that occurred between the first administration of study drug and not later than 7 days after the last administration) were reported in 39/43 (90.7%) patients overall. Most AEs (60.5%) were mild or moderate in intensity. Inhibitor development against FVIII was the most common AE/study drug-related AE, reported in 23 patients (20 of whom were PUPs); three patients (two patients with high-titer inhibitors and one patient with low-titer inhibitors) were receiving on-demand treatment at the time when inhibitors first developed. Six patients developed transient, low-titer inhibitors that resolved within 6 months of initial detection. Eighteen of the 23 patients who developed inhibitors (all 17 patients with high-titer inhibitors and one patient with low-titer inhibitors) discontinued from the main study, while the remaining five, all of whom had low-titer inhibitors, remained. Four of these five patients were on prophylaxis with BAY 81–8973 at the time of inhibitor development, and the dose and/or dosing frequency was increased in three patients following the detection of inhibitors ( Supplementary Table S5 [available in the online version]). Inhibitor development also accounted for all four study drug discontinuations that occurred due to AEs. In all cases, inhibitor development was assessed as serious (as required by the study protocol) and related to the study drug, and in all but one case, inhibitors developed within the first 20 EDs (median: 9 [range: 4–42] EDs; mean: 11 [SD: 8.2] EDs). Excluding one patient who had no inhibitor measurements after study drug administration and who discontinued after 1 ED, the overall inhibitor incidence rate was 54.8% (95% confidence interval [CI]: 38.7–70.2), and the incidence of high-titer inhibitors was 40.5% (95% CI: 25.6–56.7) ( Fig. 2 ). Among patients who developed high-titer inhibitors ( *N* = 17), peak inhibitor titers remained <10 BU/mL in three patients (including the period of ITI treatment in the extension study). Among 23 patients who developed an inhibitor (20 PUPs, 3 MTPs [two of whom had been previously treated with rFVIII products and one of whom had previously received a plasma-derived FVIII product]), 15 had mutations known to be high risk for inhibitor development, 1 had a low-risk mutation, and 7 could not be evaluated due to insufficient data or lack of consent ( Supplementary Table S6 [available in the online version]). In addition to family history of inhibitor development, 12 of 14 patients with high-titer inhibitor and available FVIII mutation data had high-risk mutations for inhibitor development.

Other frequent AEs were pyrexia (13 [30.2%] patients), nasopharyngitis (6 [14.0%]), and diarrhea (6 [14.0%]). Overall, 26 (60.5%) patients experienced at least one SAE; in most patients, these SAEs were development of a FVIII inhibitor (22 patients with an SAE of “anti-factor VIII antibody positive” and one patient with an SAE of “factor VIII inhibition”). The patient with the TEAE of “hemarthrosis” also had the TEAE of “FVIII antibody positive.” All instances of inhibitor development, and a hemarthrosis in one patient, were considered related to the study drug. There were no deaths, no intracranial hemorrhages, and no clinically relevant changes in vital signs or laboratory values.

#### Outcome of ITI in Patients with Inhibitors

Patients in the LEOPOLD Kids study who developed inhibitors were offered the option of receiving ITI treatment in the LEOPOLD Kids extension phase. Fourteen patients with high-titer inhibitor and 6 patients with low-titer inhibitor rolled over into the extension phase; of these, 12 received ITI (11 patients with high-titer inhibitor and 1 with low-titer inhibitor). The protocol-recommended dosage for ITI was a maximum of 100 IU/kg twice daily or 200 IU/kg once daily, at the discretion of the investigator and coordinating investigator, according to the clinical needs of the patient. Five patients received a high-dose ITI regimen (100 IU/kg twice daily or 200 IU/kg once daily), three received a low-dose regimen (25–50 IU/kg three times per week or every other day), and the remaining four patients were treated with an intermediate-dose regimen. Among these 12 patients, ITI was initiated between 1 month and approximately 18.5 months after initial inhibitor detection. In patients with high-titer inhibitor, ITI initiation could be delayed until inhibitor levels decreased to 10 BU/mL.

Five of 11 patients with high-titer inhibitor and one patient with low-titer inhibitor completed ITI treatment with a negative inhibitor result ( Table 4 ). The median time from the start of ITI treatment to the first negative inhibitor result was approximately 14 months (range: 5.7–23 months) for the five patients with high-titer inhibitor. These five patients had peak inhibitor titers of 5.8, 14.0, 13.0, 89.0, and 7.6 BU/mL, respectively. The patient with low-titer inhibitor had a negative inhibitor result after 76 EDs.

**Table 4:** Summary of ITI treatment in patients with inhibitors who received ITI during the LEOPOLD Kids extension phase

Patient	Peak inhibitor titer (BU/mL)	Inhibitor titer at last measurement (BU/mL)	ITI regimen at the time of study completion/discontinuation	Duration of ITI treatment (mo)
*Patients who successfully completed ITI*				
1	5.8	<0.2	75 IU/kg every other day	8
2	14	<0.2	70 IU/kg every other day	26
3	13	<0.2	55 IU/kg every other day	17
4	89	0.5	125 IU/kg 3 ×/week	19
5	7.6	<0.2	50 IU/kg every other day	11
6 a	>2	<0.2	40 IU/kg every other day	19
*Patients who discontinued ITI* b				
1	192	85	200 IU/kg every day	10
2	1536	50	65 IU/kg 3 ×/week	17
3	35	35	75 IU/kg 3 ×/week	7
4	1164	810	100 IU/kg 3 ×/week	17
5	735	20	120 IU/kg 3 ×/week	34

Abbreviation: ITI, immune tolerance induction.

a Patient had low-titer inhibitor.

b All patients had high-titer inhibitor.

Of the six remaining patients with high-titer inhibitor who received ITI treatment in the extension phase, one (peak inhibitor titer: 72 BU/mL) had a decline in inhibitor titer to 1.2 BU/mL after 18 months of ITI treatment, but then discontinued from the extension phase due to a family decision. The other five patients discontinued from the extension with positive inhibitor status after 7 to 34 months of ITI. Four of these patients had a continued decline in inhibitor titer throughout the extension phase ( Table 4 ); all four had intercurrent events, namely device infections, bleeds, or surgery for central venous access device removal.

## Discussion

In this phase 3 clinical study, BAY 81–8973 was demonstrated to be effective for the prophylaxis and treatment of bleeds in PUPs and MTPs <6 years of age with severe hemophilia A. In patients who did not develop inhibitors, the median (IQR) ABR for bleeds within 48 hours after a prophylaxis infusion was 0.0 (0.0–1.8), and the respective mean (SD) ABR was 0.9 (1.4). The primary endpoint of bleeds within 48 hours after a prophylaxis infusion was chosen due to the variable treatment intervals required in children, for whom a lower injection frequency may be related to venous access problems. 21 Of 105 treatment-requiring bleeds that occurred during prophylaxis, 82 (78%) were successfully treated with ≤2 infusions of BAY 81–8973, and treatment response was “good” or “excellent” in almost 80% of all treated bleeds (93.8% in patients without an inhibitor). Hemostatic efficacy was also “good” or “excellent” in four of five minor surgical procedures conducted during BAY 81–8973 prophylaxis and for which hemostatic assessment was provided. These efficacy findings are consistent with the use of this class of FVIII products in this patient population and complement those reported in the LEOPOLD I and LEOPOLD II trials in adults/adolescents, as well as those reported in Part A of LEOPOLD Kids. 19 20 21

Overall, the drug-related AEs observed with BAY 81–8973 in this study were in line with those known for this class of FVIII products in this patient population (PUPs/MTPs): a large majority of AEs were nonserious and mild to moderate in severity, there were no serious unexpected AEs, and no new safety concerns were identified. Consistent with the use of FVIII replacement therapies in PUPs/MTPs, inhibitor development was the most common drug-related AE. Key observational studies of large cohorts of PUPs have reported inhibitor incidence rates of 24 to 30.8%, with high-titer inhibitors reported in 14.7 to 20.2% of patients. 27 28 29 30 In a multicenter randomized study conducted to evaluate inhibitor incidence in PUPs and MTPs treated with either plasma-derived or rFVIII products, inhibitor development was observed in 44.5% (95% CI: 34.7–54.3) of patients treated with rFVIII products ( *n* = 126), and high-titer inhibitors were detected in 28.4% (95% CI: 19.6–37.2%). 31 Studies assessing single FVIII products in PUPs/MTPs have reported inhibitor development in 19.7 to 52.0% of patients, with incidence rates of 11.7 to 21.7% for high-titer inhibitors. 32 33 34 35 36 While the incidence of inhibitors in LEOPOLD Kids Part B is higher than these previously reported rates, inhibitor incidence cannot be directly compared between studies due to inherent differences in study populations and design. For example, the detection of antibody formation is dependent on the sensitivity and specificity of the assay used, and the observed incidence of antibody (including neutralizing antibody) positivity is also affected by the assay methodology used and other factors such as sample handling, timing of sample collection, use of concomitant medications, and underlying disease. 37 38 For these reasons, it may be misleading to compare the incidence of antibodies between products without standardizing both the assays used and the way inhibitors are reported across different studies. 39 Therefore, we advocate that developers of antihemophilic products strive to be more consistent when reporting or defining inhibitors in the package inserts and monographs of their products, using the checklist provided by Kwan and colleagues 39 as a guide to the minimal information that should be included.

Further, inhibitor development is multifactorial, resulting from an intricate interplay of both genetic and environmental factors. 40 41 The most compelling patient-related factors are hemophilia severity, *F8* gene mutations, and other genetic factors such as family history of inhibitors, and race or ethnicity. 42 43 44 More than one contributing cause could influence that risk. 41 Among the 14 patients in LEOPOLD Kids Part B who developed a high-titer inhibitor and who had *F8* gene mutation data available, 12 (85.7%) had mutations known to be high risk for inhibitor development, including eight intron-22 inversions. Importantly, the inhibitor rate in LEOPOLD Kids Part B was also strongly influenced by 10 inhibitor cases (high titer, *N* = 9), considered to be a “cluster” as these events were characterized by occurrence in near-consecutively treated patients over a 6-month period (June–December 2016), in 10 centers in patients receiving different batches of study product. This cluster of events represented a distinct change in the previously observed inhibitor rate (overall: 38%, high titer: 19%) in 21 treated patients. The occurrence of this cluster prompted a temporary suspension of study enrollment to undertake a comprehensive investigation of the reasons for the cluster. Clinical evaluation of the cluster of inhibitor cases in this phase 3 study revealed that most patients had one or more identified risk factors for inhibitor development: genetic mutations and intensive treatment/high doses ( *N* = 2); intensive treatment/high doses ( *N* = 2); ethnicity ( *N* = 1); genetic mutations, intensive treatment/high doses, vaccination, and ethnicity ( *N* = 1); and genetic mutations, intensive treatment/high doses, vaccination, ethnicity, and inflammation/infection ( *N* = 1). However, no clear underlying cause was identified. Further, the pattern of inhibitor development observed after the study was re-opened for enrolment suggests that the cluster was not representative of the entire population. A similar assessment of the data was provided by the study Data Monitoring Committee, which also assessed the overall risk/benefit of BAY 81–8973 as remaining unchanged. Thus, the inhibitor frequency observed in LEOPOLD Kids Part B may be related to unidentified confounding factors or may be a chance finding. Additionally, an inherent limitation of LEOPOLD Kids Part B in providing a true estimate of the immunogenicity risk of BAY 81–8973 in PUPs is the small sample size and resultant large confidence interval (95% CI: 38.7–70.2) for the overall inhibitor development rate. In any event, product immunogenicity is better explored in studies evaluating PTPs, as PUPs are naturally at a higher risk of inhibitor development. 45 46 47

Immune tolerance induction remains the only clinically proven strategy for achieving antigen-specific tolerance to FVIII in patients who develop inhibitors. 1 Consistent with the findings of a retrospective analysis of the International Immune Tolerance Registry, 48 the German registry, 49 and the North American Immune Tolerance Registry 50 performed by DiMichele and Kroner, 51 four of the five patients with high-titer inhibitor in the current study who completed ITI treatment with a negative inhibitor test had relatively low peak inhibitor titers (5.8, 14.0, 13.0, and 7.6 BU/mL).

## Conclusion

In LEOPOLD Kids Part B, BAY 81–8973 was found to be effective for the prevention and treatment of bleeds in PUPs and MTPs <6 years of age with severe hemophilia A. Consistent with the use of this class of FVIII product in PUPs/MTPs, inhibitor development was the most frequently reported AE, albeit occurring at a slightly higher frequency than has been reported by other studies of FVIII products in PUPs/MTPs. The inhibitor incidence observed during the study was strongly influenced by a cluster of inhibitor cases and may be a chance finding or due to unknown confounding risk factors. While a clear conclusion on the immunogenicity risk of BAY 81–8973 cannot be made from the results of this study, the overall benefit and risk of treatment with BAY 81–8973 remains within expectations for rFVIII products, and is supported by ongoing, long-term safety surveillance. Finally, immune tolerance can be achieved with BAY 81–8973.

## Notes

**What is known about this topic?**

- BAY 81–8973 is a full-length, unmodified, recombinant, human factor VIII approved for the treatment of patients with hemophilia A.
- In the LEOPOLD I, LEOPOLD II, and LEOPOLD Kids Part A trials, prophylaxis with BAY 81–8973 was effective for preventing and treating bleeds in previously treated adults, adolescents, and children, with good tolerability and no inhibitor development.
- The LEOPOLD Kids Part B trial evaluated BAY 81–8973 prophylaxis in previously untreated and minimally treated children aged <6 years with severe hemophilia A.

**What does this paper add?**

- In LEOPOLD Kids Part B, the median annualized bleeding rate within 48 hours after a prophylaxis infusion was 0.0, and 78% of bleeds were successfully treated with ≤2 infusions.
- The inhibitor rate (23 patients [54.8%]) was influenced by 10 inhibitor cases in near-consecutively treated patients, and may be a chance finding or due to confounding risk factors. Six of 12 patients who underwent immune tolerance induction with BAY 81–8973 achieved negative inhibitor status.
- LEOPOLD Kids Part B shows that BAY 81–8973 effectively prevents and treats bleeds in previously untreated or minimally treated children, and that it can be used to achieve immune tolerance. The benefit–risk profile of BAY 81–8973 remains unchanged.
