# Supplementary material for: BAY 81-8973 Efficacy and Safety in Previously Untreated and Minimally Treated Children with Severe Hemophilia A: The LEOPOLD Kids Trial
Source: Thromb Haemost. 2023 Jan 10;123(1):27–39. doi: 10.1055/s-0042-1757876 (PMC9831689; doi:10.1055/s-0042-1757876)
Supplement: Supplementary file 1 — Supplementary Material [file 10-1055-s-0042-1757876-s22030113.pdf]

Supplementary Table S1 Bleeds within 48 hours after prophylaxis infusion by bleed type and inhibitor status

	Inhibitor status		
	**No inhibitors (*N* = 20)**	**Low titer[a] (*N* = 6)**	**High titer[b] (*N* = 17)**
ABR for spontaneous bleeds within 48 hours after prophylaxis infusion			
Median (IQR)	0.0 (0.0–0.0)	0.0 (0.0–0.0)	0.0 (0.0–0.0)
Mean (SD)	0.1 (0.5)	0.0 (0.0)	0.9 (2.5)
ABR for trauma bleeds within 48 hours after prophylaxis infusion			
Median (IQR)	0.0 (0.0–0.5)	0.0 (0.0–0.0)	0.0 (0.0–0.0)
Mean (SD)	0.6 (1.4)	0.8 (2.0)	1.2 (2.5)
ABR for joint bleeds within 48 hours after prophylaxis infusion			
Median (IQR)	0.0 (0.0–0.0)	0.0 (0.0–0.0)	0.0 (0.0–0.0)
Mean (SD)	0.2 (0.6)	0.0 (0.0)	0.0 (0.1)

Abbreviations: ABR, annualized bleeding rate; IQR, interquartile range; *N*, total number of patients; SD, standard deviation

[a]Patients who developed low-titer inhibitors remained in the study, apart from one patient who was removed at the investigator's discretion to initiate ITI in the extension phase. Therefore, the presented bleed data represent the entire time on prophylaxis (before and after confirmation of low-titer inhibitors).

[b]All patients who developed a high-titer inhibitor were removed from the study to initiate ITI (with the option of entering the extension phase). Therefore, bleed data for patients who developed high-titer inhibitors represent *only* the period before inhibitors were confirmed.

 © 2023. The Author(s).

Supplementary Table S2 Summary of bleeds by inhibitor status in the per protocol population

	Patients without inhibitor	Patients with low-titer inhibitor	Patients with high-titer inhibitor
**Number of patients**	**18**	**6**	**16**
Patients with ≥1 bleed, *n* (%)			
No	4 (22.2)	0	2 (12.5)
Yes	14 (77.8)	6 (100)	14 (87.5)
Patients with ≥1 bleed within 48 hours, *n* (%)			
No	10 (55.6)	4 (66.7)	8 (50.0)
Yes	8 (44.4)	2 (33.3)	8 (50.0)
ABR for total bleeds			
Median (IQR)	3.3 (1.2–6.3)	5.9 (4.3–16.2)	6.2 (2.5–8.1)
Mean (SD)	5.1 (6.1)	8.8 (7.1)	9.8 (12.5)
ABR for joint bleeds			
Median (IQR)	0.0 (0.0–1.6)	0.7 (0.0–1.9)	0.0 (0.0–2.0)
Mean (SD)	0.9 (1.3)	1.3 (1.8)	1.3 (2.3)
ABR for spontaneous bleeds			
Median (IQR)	0.0 (0.0–1.2)	2.5 (0.0–4.6)	0.0 (0.0–2.6)
Mean (SD)	1.0 (2.1)	3.8 (5.0)	2.3 (4.3)
ABR for traumatic bleeds			
Median (IQR)	1.9 (0.0–3.2)	1.4 (0.0–2.2)	2.5 (0.5–5.3)
Mean (SD)	2.0 (2.1)	1.5 (1.4)	5.5 (10.9)
ABR for total bleeds occurring within 48 hours after prophylaxis infusion			
Median (IQR)	0.0 (0.0–1.5)	0.0 (0.0–1.9)	0.2 (0.0–4.3)
Mean (SD)	0.9 (1.4)	1.4 (2.5)	2.3 (3.0)
ABR for joint bleeds occurring within 48 hours after prophylaxis infusion			
Median (IQR)	0.0 (0.0–0.0)	0.0 (0.0–0.0)	0.0 (0.0–0.0)
Mean (SD)	0.2 (0.7)	0.0 (0.0)	0.0 (0.1)
ABR for spontaneous bleeds occurring within 48 hours after prophylaxis infusion			
Median (IQR)	0.0 (0.0–0.0)	0.0 (0.0–0.0)	0.0 (0.0–0.0)
Mean (SD)	0.1 (0.5)	0.0 (0.0)	0.6 (1.6)
ABR for traumatic bleeds occurring within 48 hours after prophylaxis infusion			
Median (IQR)	0.0 (0.0–1.1)	0.0 (0.0–0.0)	0.0 (0.0–0.7)
Mean (SD)	0.7 (1.4)	0.3 (0.8)	1.1 (2.4)

Abbreviations: ABR, annualized bleeding rate; IQR, interquartile range; SD, standard deviation.
Note: Total bleeds: sum of spontaneous, traumatic, joint and "other" bleeds. Bleeds within 48 hours: bleeds within 48 hours of previous prophylaxis infusion.

 © 2023. The Author(s).

Supplementary Table S3 Summary of bleeds in the period of low-titer inhibitor[a] in patients who developed low-titer inhibitors

	Patients with low-titer inhibitor
**Number of patients**	**6**
Patients with ≥1 bleed, *n* (%)	
No	3 (50.0)
Yes	3 (50.0)
Patients with ≥1 bleed within 48 hours, *n* (%)	
No	4 (66.7)
Yes	2 (33.3)
ABR for total bleeds	
Median (IQR)	6.0 (0.0–13.7)
Mean (SD)	8.1 (9.7)
ABR for joint bleeds	
Median (IQR)	0.0 (0.0–0.0)
Mean (SD)	0.6 (1.4)
ABR for spontaneous bleeds	
Median (IQR)	0.0 (0.0–4.0)
Mean (SD)	2.4 (4.2)
ABR for trauma bleeds	
Median (IQR)	0.0 (0.0–3.4)
Mean (SD)	1.2 (1.9)
ABR for total bleeds occurring within 48 hours after prophylaxis infusion	
Median (IQR)	0.0 (0.0–3.4)
Mean (SD)	4.4 (9.3)
ABR for joint bleeds occurring within 48 hours after prophylaxis infusion	
Median (IQR)	0.0 (0.0–0.0)
Mean (SD)	0.0 (0.0)
ABR for spontaneous bleeds occurring within 48 hours after prophylaxis infusion	
Median (IQR)	0.0 (0.0–0.0)
Mean (SD)	0.0 (0.0)
ABR for traumatic bleeds occurring within 48 hours after prophylaxis infusion	
Median (IQR)	0.0 (0.0–0.0)
Mean (SD)	0.6 (1.4)

Abbreviations: ABR, annualized bleeding rate; IQR, interquartile range; SD, standard deviation.
Note: Total bleeds: sum of spontaneous, traumatic, joint and “other” bleeds. Bleeds within 48 hours: bleeds within 48 hours of previous prophylaxis infusion.
[a]Period of low-titer inhibitor started on the day when low-titer inhibitor was detected and ended with either the final visit or the day when no inhibitor was detected, whichever came first.

 © 2023. The Author(s).

Supplementary Table S4 Minor surgeries

		BAY 81–8973 dose (IU)		
Patient	Procedure	Day of surgery[a]	Total[b]	Haemostasis
1	Port insertion	750	3,000	Excellent
2	Simple frenectomy	250	250	Excellent
3	Port insertion—right external jugular	0	NR	NR
4	Vein cutdown and port insertion	1,000	4,000	Excellent
5	Port insertion	0	NR	Good

Abbreviations: IVR, intravenous reservoir; NR, not reported.
[a]Total dose (IU) of all infusions on the day of the surgery.
[b]Total dose (IU) of all surgery infusions.

Supplementary Table S5 Patients with low-titer inhibitors who completed the main study and entered the extension phase

Patient	PUP/MTP	Age at enrollment	Exposure days at first inhibitor detection	Inhibitor titer at first detection (BU/mL)	Treatment at the time of first inhibitor detection	Prophylaxis dose before inhibitor development	Prophylaxis dose after inhibitor development
1	PUP	11 mo	9	0.6	On demand	N/A[a]	N/A[a]
2	PUP	10 mo	10	3.8	Prophylaxis	25 IU/kg once weekly	45 IU/kg twice weekly
3	MTP	2 y	16	<5	Prophylaxis	25 IU/kgonce weekly	Unchanged
4	PUP	1 y	38	0.8	Prophylaxis	25 IU/kgonce weekly	25 IU/kg three times weekly
5	PUP	10 mo	15	1.3	Prophylaxis	25 IU/kgonce weekly	25 IU/kg twice weekly, then 50 IU/kg three times weekly

Abbreviations: MTP, minimally treated patient; N/A, not applicable; PUP, previously untreated patient.
[a]Patient started regular prophylaxis with BAY 81–8973 approximately 1 month after first detection of FVIII inhibitor, at a dose of 40 IU/kg every 3 days; 16 days later the dosing frequency was increased to three times per week. This dose was not changed when inhibitors reoccurred ∼34 days later.

 © 2023. The Author(s).

Supplementary Table S6 Genetic mutation analysis in patients who developed inhibitors

Patient	Inhibitor titer	MTP/PUP	High- or low-risk mutation[a]	Genetic mutations provided by investigator	HGVS nucleotide	HGVS amino acid	Variant type	Variant effect/polymorphism or pathogenic
**1**	**High**	**PUP**	**High**	**–**	**c.3780C > G**<br>**c.6769A > G**<br>**Intron 22 inversion**	**p.(Asp1260Glu)**<br>**p.(Met2257Val)**	**Substitution**<br>**Substitution**<br>**Inversion**	**Missense/polymorphism**<br>**Missense/polymorphism**<br>**Inversion 22/pathogenic**
2	Low	PUP	High	–	c.3780C > G<br>c.1010–27G > A<br>c.4042_4045delAAAA	p.(Asp1260Glu)<br>n.a.<br>(p.Lys1348GlyfsTer2)	Substitution<br>Substitution<br>Deletion	Missense/polymorphism<br>Intron/polymorphism<br>Frameshift/pathogenic
3	High	PUP	High	–	Intron 22 inversion		Inversion	Inversion 22/pathogenic
4	High	PUP	High	–	c.3864A > C<br>c.5831_5841del	p.(Ser1288Ser)<br>(p.Ile1944ThrfsTer23)	Substitution<br>Deletion	Silent/polymorphism<br>Frameshift/pathogenic
**5**	**High**	**PUP**	**High**	**–**	**c.389–9C > T**<br>**Intron 22 inversion**	**n.a.**	**Substitution**<br>**Inversion**	**Intron/polymorphism**<br>**Inversion 22/pathogenic**
**6**	**High**	**MTP**	**High**	**–**	**c.4076G > A**<br>**c.3864A > C**	**p.(Trp1359*)**<br>**p.(Ser1288Ser)**	**Substitution**<br>**Substitution**	**Nonsense/pathogenic**<br>**Silent/polymorphism**
7	Low	PUP	High	–	Intron 22 inversion		Inversion	Inversion 22/pathogenic
**8**	**High**	**PUP**	**Low**	**–**	**c.2945dupA**	**p.(Asn982Lysfs*9)**	**Duplication**	**Frameshift/pathogenic**
9	Low	PUP	[b]	Splice mutation[c]				
10	High	PUP	[b]	–				
**11**	**High**	**PUP**	**Not applicable[d]**	**–**				
12	High	PUP	High	–	Intron 22 inversion		Inversion	Inversion 22/pathogenic
13	Low	MTP	[b]	–	c.3864A > C	p.(Ser1288Ser)	Substitution	Silent/polymorphism
14	High	PUP	High		c.3864A > C<br>c.221delC	p.(Ser1288Ser)<br>p.(Thr74Argfs*18)	Substitution<br>Deletion	Silent/polymorphism<br>Frameshift/pathogenic
15	High	MTP	High	Nonsense mutation[c]				
16	Low	PUP	High	–	c.2440C > T	p.(Arg814*)	Substitution	Nonsense/pathogenic
**17**	**High**	**PUP**	**Not applicable[d]**	**–**				
**18**	**High**	**PUP**	**High**	**–**	**c.3780C > G**<br>**c.1010–27G > A**<br>**Intron 22 inversion**	**p.(Asp1260Glu)**<br>**n.a.**	**Substitution**<br>**Substitution**<br>**Inversion**	**Missense/polymorphism**<br>**Intron/polymorphism**<br>**Inversion 22/pathogenic**
**19**	**Low**	**PUP**	**Not applicable[d]**	**–**				
20	High	PUP	High	–	c.3780C > G<br>Intron 22 inversion	p.(Asp1260Glu)	Substitution<br>Inversion	Missense/polymorphism<br>Inversion 22/pathogenic

 © 2023. The Author(s).

Supplementary Table S6 (*Continued*)

Patient	Inhibitor titer	MTP/PUP	High- or low-risk mutation[a]	Genetic mutations provided by investigator	HGVS nucleotide	HGVS amino acid	Variant type	Variant effect/polymorphism or pathogenic
**21**	**High**	**PUP**	**High**	–	**c.3780C > G<br>c.1010–27G > A<br>c.6345delC**	**p.(Asp1260Glu)<br>n.a.<br>(p.Tyr2116ThrfsTer27)**	**Substitution<br>Substitution<br>Deletion**	**Missense/polymorphism<br>Intron/polymorphism<br>Frameshift/pathogenic**
22	High	PUP	[b]	–	c.3780C > G<br>c.1010–27G > A	p.(Asp1260Glu)<br>n.a.	Substitution<br>Substitution	Missense/polymorphism<br>Intron/polymorphism
**23**	**High**	**PUP**	**High**	–	**c.(520 + 1_521–1)_(723 + 1_724–1)del<br>Intron 22 inversion**	**Del exon 6**	**Deletion<br>Inversion**	**Large structural change (>50 bp)<br>Inversion 22/pathogenic**

Abbreviations; MTP, minimally treated patient; PUP, previously untreated patient.
Note: Patients highlighted in **bold** comprise the cluster of 10 inhibitor cases that occurred in the middle of the study.
[a]Large deletions, nonsense mutations, and intron-22 inversions carry a higher risk of inhibitor development than small deletions and insertions, missense mutations, and splice-site mutations [Schwaab et al., 1995; Gouw et al., 2013a]. As no in-depth genetic analysis was performed, this categorization serves exploratory purposes only.
[b]"Insufficient data" was used in cases where neither historical data nor central laboratory analyses were available (not collected or mutation not identified).
[c]Only historical data (as provided by the Investigator) are available.
[d]"Not applicable" was used for participants lacking informed consent for pharmacogenetic studies.

 © 2023. The Author(s).
